# A Genetic Risk Variant Associated With the Risk of Primary Biliary Cholangitis Is Inherited From Neanderthals

**DOI:** 10.1111/liv.70868

**Published:** 2026-09-22

**Authors:** Alessio Gerussi, Chiara Caime, Harold Wang, Heather J. Cordell, George F. Mells, Richard N. Sandford, David E. Jones, Gideon Hirschfield, Katherine A. Siminovitch, M. Eric Gershwin, Brian D. Juran, Elizabeth J. Atkinson, Angela Cheung, Mariza de Andrade, Aris Baras, Konstantinos N. Lazaridis, Rosanna Asselta, Pietro Invernizzi, Manuela Sironi, Sriram Sankararaman, Canadian‐US PBC Consortium, UK‐PBC Consortium, James J. Fryett, Rebecca Darlay, Steven Flack, Ann Spicer, Victoria L. Mulcahy, Richard Sturgess, Christopher Healey, Andrew Yeoman, Anton Vj Gunasekera, Paul Kooner, Kapil Kapur, V. Sathyanarayana, Yiannis Kallis, Javaid Subhani, Rory Harvey, Roger McCorry, Paul Rooney, David Ramanaden, Richard Evans, Thiriloganathan Mathialahan, Jaber Gasem, Christopher Shorrock, Mahesh Bhalme, Paul Southern, Jeremy A. Tibble, David A. Gorard, Susan Jones, Brijesh Srivastava, Matthew R. Foxton, Carole E. Collins, David Elphick, Mazn Karmo, Francisco Porras‐Perez, Michael Mendall, Tom Yapp, Minesh Patel, Roland Ede, Joanne Sayer, James Jupp, Neil Fisher, Martyn J. Carter, Konrad Koss, Jayshri Shah, Andrzej Piotrowicz, Glyn Scott, Charles Grimley, Ian R. Gooding, Simon Williams, Judith Tidbury, Guan Lim, Kuldeep Cheent, Sass Levi, Dina Mansour, Matilda Beckley, Coral Hollywood, Terry Wong, Richard Marley, John Ramage, Harriet M. Gordon, Jo Ridpath, Theodore Ngatchu, Vijay Paul Bob Grover, Ray G. Shidrawi, George Abouda, L. Corless, Mark Narain, Ian Rees, Ashley Brown, Simon Taylor‐Robinson, Joy Wilkins, Leonie Grellier, Paul Banim, Debasish Das, Michael A. Heneghan, Howard Curtis, Helen C. Matthews, Faiyaz Mohammed, Mark Aldersley, Raj Srirajaskanthan, Giles Walker, Alistair McNair, Amar Sharif, Sambit Sen, George Bird, Martin I. Prince, Geeta Prasad, Paul Kitchen, Adrian Barnardo, Chirag Oza, Nurani N. Sivaramakrishnan, Prakash Gupta, Amir Shah, Chris Dj Evans, Subrata Saha, Katharine Pollock, Peter Bramley, Ashis Mukhopadhya, Stephen T. Barclay, Natasha McDonald, Andrew J. Bathgate, Kelvin Palmer, John F. Dillon, Simon M. Rushbrook, Robert Przemioslo, Chris McDonald, Andrew Millar, Cheh Tai, Stephen Mitchell, Jane Metcalf, Syed Shaukat, Mary Ninkovic, Udi Shmueli, Andrew Davis, Asifabbas Naqvi, Tom Jw Lee, Stephen Ryder, Jane Collier, Howard Klass, Matthew E. Cramp, Nichols Sharer, Richard Aspinall, Deb Ghosh, Andrew C. Douds, Jonathan Booth, Earl Williams, Hyder Hussaini, John Christie, Steven Mann, Douglas Thorburn, Aileen Marshall, Imran Patanwala, Aftab Ala, Julia Maltby, Ray Matthew, Chris Corbett, Sam Vyas, Saket Singhal, Dermot Gleeson, Sharat Misra, Jeff Butterworth, Keith George, Tim Harding, Andrew Douglass, Harriet Mitchison, Simon Panter, Jeremy Shearman, Gary Bray, Michael Roberts, Graham Butcher, Daniel Forton, Zahid Mahmood, Matthew Cowan, Debashis Das, Chin Lye Ch'ng, Mesbah Rahman, Gregory C. A. Whatley, Emma Wesley, Aditya Mandal, Sanjiv Jain, Stephen P. Pereira, Mark Wright, Palak Trivedi, Fiona H. Gordon, Esther Unitt, Altaf Palejwala, Andrew Austin, Vishwaraj Vemala, Allister Grant, Andrew D. Higham, Alison Brind, Ray Mathew, Mark Cox, Subramaniam Ramakrishnan, Alistair King, Simon Whalley, Jocelyn Fraser, S. J. Thomson, Andrew Bell, Voi Shim Wong, Richard Kia, Ian Gee, Richard Keld, Rupert Ransford, James Gotto, Charles Millson, Italian PBC Genetics Study Group, Andrea Affronti, Maurizia Brunetto, Barbara Coco, Giancarlo Spinzi, Gianfranco Elia, Carlo Ferrari, Ana Lleo, Luigi Muratori, Paolo Muratori, Piero Portincasa, Agostino Colli, Savino Bruno, Guido Colloredo, Francesco Azzaroli, Pietro Andreone, MariaConsiglia Bragazzi, Domenico Alvaro, Vincenzo Cardinale, Nora Cazzagon, Annarosa Floreani, Cristina Rigamonti, Floriano Rosina, Marco Carbone, Laura Cristoferi, Marco Festa, Eugenia Nofit, Miki Scaravaglio, Daphne D'Amato, Federica Malinverno, Pietro Lampertico, Francesca Donato, Stefano Fagiuoli, Piero L. Almasio, Edoardo Giannini, Carmela Cursaro, Massimo Colombo, Luca Valenti, Luca Miele, Angelo Andriulli, Grazia A. Niro, Ignazio Grattagliano, Lorenzo Morini, Giovanni Casella, Maria Vinci, Pier Maria Battezzati, Andrea Crosignani, Massimo Zuin, Alberto Mattalia, Vincenza Calvaruso, Silvia Colombo, Antonio Benedetti, Marco Marzioni, Andrea Galli, Fabio Marra, Mirko Tarocchi, Antonio Picciotto, Filomena Morisco, Luca Fabris, Lory Saveria Crocè, Claudio Tiribelli, Pierluigi Toniutto, Mario Strazzabosco

**Affiliations:** ^1^ Department of Medicine and Surgery University of Milano‐Bicocca Monza Italy; ^2^ Division of Gastroenterology, Center for Autoimmune Liver Diseases European Reference Network on Hepatological Diseases (ERN RARE‐LIVER), Fondazione IRCCS San Gerardo Dei Tintori Monza Italy; ^3^ Department of Computer Science University of California Los Angeles California USA; ^4^ Population Health Sciences Institute, Faculty of Medical Sciences Newcastle University UK; ^5^ Academic Department of Medical Genetics Cambridge University Cambridge UK; ^6^ Faculty of Medical Sciences Newcastle University Newcastle UK; ^7^ The Autoimmune and Rare Liver Disease Programme, Division of Gastroenterology and Hepatology Toronto General Hospital Toronto Ontario Canada; ^8^ Department of Medicine University of Toronto Toronto Ontario Canada; ^9^ Mount Sinai Hospital, Lunenfeld Tanenbaum Research Institute and Toronto General Research Institute Toronto Canada; ^10^ Department of Immunology University of Toronto Toronto Ontario Canada; ^11^ Institute of Medical Sciences University of Toronto Toronto Ontario Canada; ^12^ University of California – Davis Davis California USA; ^13^ Division of Gastroenterology and Hepatology Mayo Clinic Rochester Minnesota USA; ^14^ Division of Biomedical Statistics and Informatics Mayo Clinic Rochester Minnesota USA; ^15^ Regeneron Genetics Center Tarrytown New York USA; ^16^ Department of Biomedical Sciences Humanitas University Milan Italy; ^17^ IRCCS Humanitas Research Hospital Milan Italy; ^18^ Scientific Institute IRCCS E. MEDEA, Bioinformatics Bosisio Parini Italy; ^19^ Department of Computational Medicine University of California Los Angeles California USA; ^20^ Department of Human Genetics University of California Los Angeles California USA

## Abstract

**Background:**

Primary Biliary Cholangitis (PBC) is an autoimmune cholangiopathy with polygenic architecture and unknown aetiology. Evidence links Neanderthal‐derived genetic variants to autoimmune conditions; however, their contribution to PBC susceptibility remains unexplored.

**Objective:**

We investigate whether archaic alleles influence PBC risk.

**Design:**

Genotype data from four PBC cohorts were analysed: CANUK (4615 cases, 9233 controls), OLD IT (444 cases, 901 controls), NEW IT (255 cases, 579 controls) and MAYO (891 cases, 621 controls). Association testing with 235 592 Neanderthal Informative Markers (NIMs) was performed, adjusting for population stratification. Heritability was estimated using RHE‐mc and temporal haplotype dynamics were assessed using ancient DNA data from Europe and Asia.

**Results:**

A Neanderthal‐derived variant in *TNPO3* (rs12531711), previously reported by Cordell et al. showed strong association in CANUK (*p* = 2.04 × 10^−26^) and replicated across all validation cohorts. This variant functions as an eQTL, upregulating *IRF5* and downregulating *TNPO3* in blood. It is rare in African populations but common both in Europeans and Asians. Temporal analysis of the haplotype carrying rs12531711 revealed frequency increased substantially during the Bronze Age (5000–3000 BP), rising from 2.0% to 7.7% in Europe and 3.4% to 17.4% in Asia, before stabilizing. Despite this strong single‐variant association, the heritability explained by NIMs was not significantly different from that of non‐NIM variants.

**Conclusion:**

A Neanderthal‐derived variant contributes to PBC risk and its frequency increased in the Bronze Age, possibly due to pathogen‐driven selection. Archaic introgression overall has limited influence on PBC heritability. Functional studies are needed to elucidate the role of rs12531711.

## Key Points

**What Is Already Known on This Subject?:**

- Primary biliary cholangitis is a complex autoimmune liver disease with established genetic risk loci.
- Neanderthal‐derived variants have been associated with immune‐related traits, but their collective contribution to PBC genetic architecture has not been systematically evaluated.

**What Are the New Findings?:**

- Temporal genetic analysis reveals a Neanderthal‐derived PBC risk variant (rs12531711) markedly increased during the Bronze Age across Eurasia.
- Despite strong single‐locus effects, Neanderthal variants collectively contribute minimally to PBC genetic architecture, suggesting most introgressed alleles have negligible disease impact.

**How Might It Impact on Clinical Practice in the Foreseeable Future?:**

- These findings enhance understanding of the genetic architecture underlying PBC by identifying evolutionary origins of immune dysregulation.
- While, given its high population frequency, utility for clinical risk prediction is limited, characterising the role of rs12531711 in IRF5‐mediated immune activation may inform future therapeutic strategies targeting interferon pathways.

## Lay Summary

Neanderthals are extinct hominins that interbred with ancestors of modern humans, and genetic variants of Neanderthal origin still persist in present‐day humans and play a role in human disease. This study shows that one variant of Neanderthal origin is associated with the genetic risk of developing a rare liver disease called primary biliary cholangitis.

## 1 Introduction

Neanderthals, an extinct hominin group, inhabited Eurasia prior to the migration of anatomically modern humans out of Africa. They are regarded as the closest relatives of present‐day humans from an evolutionary perspective [1]. It is now established that Neanderthal genome segments persist in modern human genomes and may influence susceptibility to diseases, in particular to those due to immune‐related abnormalities [2]. The first indications of interbreeding between Neanderthals and modern humans surfaced in 2010 through a study comparing Neanderthals and present‐day human genomes, revealing notable genetic overlap in Eurasian populations compared to those from Sub‐Saharan Africa. Subsequent research estimated that 1%–4% of the non‐African human genome, primarily from Eurasian populations, originated from Neanderthals and that admixture likely occurred between 40 000 and 54 000 years before present (BP) [3, 4, 5, 6, 7].

Beyond their significance in evolutionary anthropology, these findings have intriguing implications for human health, suggesting that a subset of alleles inherited from ancient hominins have conferred either advantageous or disadvantageous traits to modern humans. For instance, introgressed archaic alleles are thought to have contributed substantial immune advantages to modern humans. A notable example is a cluster of three Toll‐like receptor (TLR) genes, whose archaic alleles have been shown to influence TLR gene expression levels. These alleles are linked to heightened microbial resistance, yet they are also associated with an increased prevalence of allergic diseases, as observed in large‐scale cohort studies [8]. Additionally, evidence suggests that Neanderthal DNA contributed to the adaptation of modern humans against viruses [9]. However, archaic alleles can exert an influence on susceptibility to a spectrum of diseases. For example, recent research shed light on the impact of Neanderthal‐derived haplotypes on vulnerability to type 2 diabetes. The identification of genetic variants within the *SLC16A11* gene as factors contributing to the risk of type 2 diabetes in individuals of Mexican descent highlights the key role played by genetic variants introduced by Neanderthals in the predisposition to metabolic disorders [10]. Furthermore, emerging evidence suggested a broader association of Neanderthal‐derived genetic variants with autoimmune conditions such as rheumatoid arthritis and systemic lupus erythematosus, offering new insights into the genetic basis of autoimmune diseases [10]. In general, archaic alleles were shown to have contributed significantly to the genetic makeup of immune‐mediated and metabolic traits [2].

Among autoimmune diseases, primary biliary cholangitis (PBC) stands out as a rare liver condition with a complex genetic architecture. PBC is characterized by progressive impairment of the intrahepatic bile ducts, which leads to fibrosis and eventually cirrhosis. It is marked by cholestasis, positivity for anti‐mitochondrial (AMA) or antinuclear (ANA) antibodies and histological evidence of granulomatous lymphocytic small bile duct cholangitis, with a notable female predominance (female‐to‐male ratio up to 9:1) [11, 12, 13]. The aetiology of the disease is still unknown, but several studies suggested the crucial role of both genetic and environmental factors. Genome‐wide association studies (GWAS) investigating the genetic basis of PBC have revealed clear associations with the *HLA* region and several non‐*HLA* loci [14, 15]. Particularly intriguing is the X chromosome, which harbours several genes involved in immune functions and exhibits a higher frequency of loss in female patients with PBC [16, 17, 18].

Despite these findings, the role of Neanderthal alleles in shaping susceptibility to PBC remains unexplored, offering a novel direction for uncovering insights into its genetic architecture. In this study, we investigated whether Neanderthal‐derived alleles contribute to the risk of developing PBC.

## 2 Material and Methods

### 2.1 Participants and Genotype Data

This study included genotype data from previously published GWASs [19, 20, 21, 22]. Four case–control cohorts of European ancestry were included:

- a combined cohort of participants from Canada and the United Kingdom (4615 cases and 9233 controls), from here on referred to as CANUK;
- an Italian cohort from Northern Italy (444 cases and 901 controls), from here on referred to as OLD_IT;
- an Italian cohort from Central and Southern Italy (255 cases and 579 controls), from here on referred to as NEW_IT;
- a cohort from the Mayo Clinic in the United States (891 cases and 621 controls), from here on referred to as MAYO.

All cases were confirmed to meet internationally recognized diagnostic criteria for PBC [23]. Written informed consent for genetic analysis was obtained from all participants and the respective study protocols received approval from local institutional review boards.

Quality control was performed separately for each dataset in a stepwise manner. Individuals were excluded if they exhibited cryptic relatedness (PI_Hat > 0.10), > 10% missing genotypes or sex discrepancies based on X chromosome heterozygosity. Single‐nucleotide polymorphisms (SNPs) were excluded if they had > 10% missingness, minor allele frequency (MAF) < 0.005, Hardy–Weinberg equilibrium in control females (*p*‐value < 1 × 10^−4^), significant MAF differences between control males and females (*p*‐value < 0.05/number of SNPs) or differential missingness by sex (*p*‐value < 1 × 10^−4^). The subsequent imputation and quality control procedures, described in detail in Asselta et al. [24], resulted in a final set of 8 656 760, 13 113 694, 9 964 354 and 9 964 354 SNPs for the CANUK, OLD_T, NEW_IT and MAYO cohorts, respectively.

### 2.2 Identification of ‘Neanderthal Informative Markers’ (NIMs)

We extracted a set of 235 592 SNPs (taken from Wei et al., 2023 [25]) previously identified in the 1000 Genomes project that are likely to be Neanderthal‐derived (NIMs) and examined their association with PBC in the CANUK cohort.

### 2.3 Covariates

In our analysis, we utilized 10 genotypic principal components (PCs) and 5 NIM PCs. The 10 genotypic PCs represent the first 10 PCs derived from the genotype matrix of each cohort. The inclusion of 5 NIM PCs is intended to account for stratification at NIMs that may not be adequately corrected by the genotypic PCs alone. These NIM PCs are also estimated from the cohort‐specific genotype matrices, but they are subsetted to include only NIMs.

### 2.4 Association Analysis

For our NIM association analysis, we tested 8 656 760 SNPs. We conducted the analysis separately for each population. The association testing was performed twice: first with only 10 genotypic PCs and then with an additional 5 NIM PCs. GWAS quality control was performed using the R GWASTools package [26, 27, 28].

### 2.5 Heritability Analysis

For the heritability analysis, we focused solely on the CANUK cohort. We first filtered out loci with a MAF of < 0.0001 and excluded the major histocompatibility complex (MHC) region, resulting in 8 524 781 SNPs. We then ran RHE‐mE [29], based on the RHE‐mC framework of Pazokitoroudi et al. [29], a method that partitions genetic variance across large sample sizes to estimate NIM heritability. This was done twice: once including 10 genotypic PCs for covariance adjustment and again using the five NIM PCs. In both instances, we used 10 random vectors and 100 jackknife blocks.

### 2.6 Temporal Dynamics of the Neanderthal‐Derived TNPO3 Haplotype

Linkage disequilibrium (LD) between rs10488631 and rs12531711 was evaluated using the LDpair tool (GRCh38 high coverage mode) from the LDlink suite [30].

To analyse the frequency of the Neanderthal rs10488631 allele over time, we used a compilation of ancient DNA genome data made available from the Allen Ancient DNA Resource (AADR, V54.1.p1 [Dataverse 8.0] March 6 2023) (https://doi.org/10.7910/DVN/FFIDCW, [31]). Because ancient DNA sequences often have a very low coverage and a relatively high genotyping error rate, data are pseudohaploidized by sampling one allele per variant site. Out of 9990 ancient samples, 4224 covered the rs10488631 variant. After removing related subjects we remained with data from 3354 individuals, who were assigned to continents based on geographic coordinates or country of sampling. The boundary between Europe and Asia was set at a longitude of 45°. Due to the sample size and because interbreeding with Neanderthals/Denisovans occurred in Eurasia, only samples from Asia and Europe were analysed [3, 32]. We excluded individuals from the Middle East (Israel, Lebanon, Jordan and Syria) for these analyses.

## 3 Results

### 3.1 A Neanderthal‐Derived Variant in TNPO3 Is Associated With PBC

**FIGURE 1:**
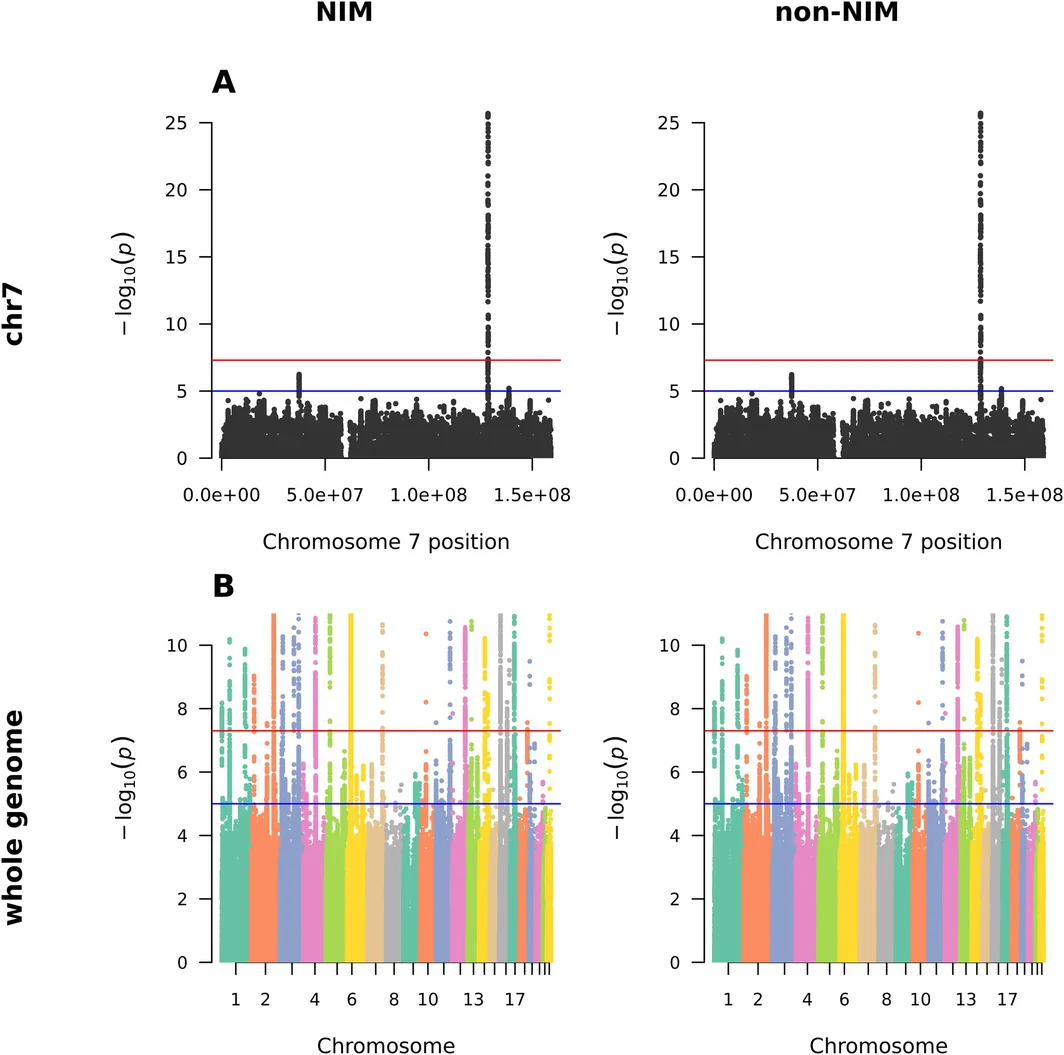
Genome‐wide association results for PBC. (A) Regional Manhattan plots showing the association of rs12531711 on chromosome 7 with and without inclusion of Neanderthal‐informative marker (NIM) principal components (PCs). (B) Genome‐wide Manhattan plots of PBC association results with (1) and without (2) inclusion of NIM PCs. The inclusion of NIM PCs does not affect the significance of the signal at rs12531711.

Consistent with previous findings from the 2021 GWAS of PBC [19], which first reported rs12531711 among the top associated loci, we confirmed and further characterized this variant in the CANUK cohort. We first tested GWAS on the 8 656 760 SNPs in the CANUK cohort for association with PBC by performing a logistic regression (Table 1; Figure 1A; Figure S1), controlling for the first 10 PCs of ancestry. We found a NIM (rs12531711) on chromosome 7 significantly associated with PBC at a *p*‐value = 1.94 × 10^−26^ (Table 1; Figure 1A). Detailed materials can be found in the Supporting Information. The association remained highly significant also after including the first 10 common PCs and 5 NIM PCs in the analysis (*p*‐value = 2.04 × 10^−26^) (Figure 1B). We then validated the association of this particular NIM by replicating this signal in three additional case–control cohorts (MAYO, OLD_IT, NEW_IT) (Table 1). Effect sizes were consistent across all cohorts, with odds ratios ranging from 1.44 to 1.65, and the direction of effect matching that observed in the discovery cohort. Replication was considered statistically significant at *p*‐value < 0.05; all three cohorts met this threshold.

**TABLE 1:** Association results for the Neanderthal‐derived variant rs12531711 across four cohorts, using 10 genotypic PCs and 5 NIM PCs.

Cohort	Chromosome	Position	Ref	Alt	Model	*N*	OR	SE (log OR)	*Z*	*p*
CANUK	7	128 617 466	A	G	ADD	13 809	1.50	0.038	10.64	2.04e‐26
OLD_IT	7	128 617 466	A	G	ADD	1344	1.49	0.131	3.04	2.34e‐03
NEW_IT	7	128 617 466	A	G	ADD	834	1.65	0.222	2.25	2.43e‐02
MAYO	7	128 617 466	A	G	ADD	1507	1.44	0.117	3.16	1.59e‐03

*Note:* Shown are the chromosomal location, reference (Ref) and alternative (Alt) alleles, statistical model used (Model), sample size (*N*), odds ratio (OR), standard error (SE) of the log(OR), *Z*‐statistic (*Z*) and *p*‐value. The association is consistently replicated across all cohorts.

**FIGURE 2:**
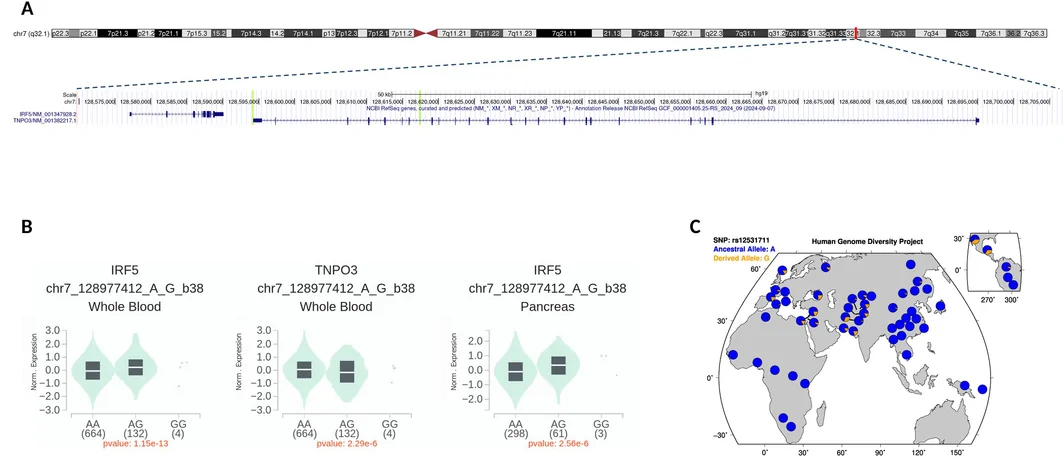
Function and geographic distribution of the Neanderthal allele at rs12531711. (A) Location of the two Neanderthal‐derived SNPs within the UCSC Genome Browser view. (B) Violin plots representing the effect of rs12531711 on the expression of *TNPO3* and *IRF5* in whole blood and pancreas. Data derived from the GTEx Portal [33]. (C) Allele frequency of rs12531711 in different human populations. Data derived from the Human Genome Diversity Panel dataset [34].

The rs12531711 SNP is an intronic variant in the *TNPO3* gene (coding for Transportin 3), mapping at chr7:128617466 (genome reference: GRCh37) (Figure 2A). The Neanderthal‐derived allele, G, increases the risk for PBC and has also been associated with other autoimmune diseases, such as systemic lupus erythematosus (LES) and rheumatoid arthritis (RA) (Table 2).

**TABLE 2:** Reported associations of the Neanderthal‐derived SNP rs12531711 with complex traits (GWAS Atlas).

Trait	Domain	*p*	Sample size	Effect allele	Non_effect allele	Reference
Systemic lupus erythematosus	Skeletal	3.5e‐52	10 995	NA	NA	29 848 360
Systemic lupus erythematosus	Skeletal	3.3e‐45	14 267	G	A	26 502 338
Primary biliary cholangitis	Gastrointestinal	5.7e‐23	13 239	G	A	26 394 269
Rheumatoid arthritis	Connective tissue	1.2e‐11	58 284	G	A	24 390 342
Rheumatoid arthritis	Connective tissue	1.2e‐11	103 638	G	A	24 390 342
Systemic lupus erythematosus	Skeletal	2.3e‐11	3094	A	G	18 204 098
Estimated glomerular filtration rate	Metabolic	3.7e‐09	567 460	A	G	31 152 163
Systemic lupus erythematosus	Skeletal	1.2e‐08	2431	A	G	29 848 360
Mouth/teeth dental problems_ mouth ulcers	Body structures	1.0e‐08	385 026	G	A	31 427 789
Estimated glomerular filtration rate	Metabolic	1.1e‐09	765 348	A	G	31 152 163

*Note:* Traits include autoimmune and metabolic conditions such as systemic lupus erythematosus (SLE), rheumatoid arthritis (RA) and estimated glomerular filtration rate (eGFR). The table reports the corresponding phenotypic domain, *p*‐values (in scientific notation), sample sizes, allelic information and source reference IDs.

Based on GTEx data (https://gtexportal.org), rs12531711 is an expression quantitative trait locus (eQTL) for both the *IRF5* gene (coding for Interferon regulatory factor 5) and *TNPO3*. Specifically, the Neanderthal‐derived PBC risk allele (rs12531711‐G) is associated with higher expression of *IRF5* in several tissues, including whole blood and pancreas; conversely, the same allele decreases *TNPO3* expression in blood (Figure 2B). Allele nomenclature for rs12531711 in Figure 2B reflects the reverse complement strand orientation (T and C), which correspond to the ancestral (A) and Neanderthal‐derived (G) alleles on the forward strand, respectively.

The Neanderthal‐derived allele of rs12531711 has variable frequency in human populations and, as expected, is virtually absent in African individuals (Figure 2C). In European populations, the allele frequency of G is approximately 10.1% with genotype frequencies of 80.9% A|A, 17.9% A|G and 1.2% G|G (*N* = 504). In South Asian populations, the G allele frequency is 13.4%, with genotype frequencies of 75.3% A|A, 22.7% A|G and 2.0% G|G (*N* = 489) (Data are from the 1000 Genomes Project).

### 3.2 Functional Annotation Using RegulomeDB

To further investigate the regulatory role of rs12531711, we performed functional annotation using RegulomeDB v2. rs 12 531 711 receives a RegulomeDB score of 1f, indicating strong evidence of regulatory activity. The variant overlaps multiple regulatory peaks in immune‐related tissues, consistent with its observed effect on *IRF5* expression (Figure S2).

### 3.3 Temporal Dynamics of the TNPO3 Neanderthal‐Derived Haplotype

**FIGURE 3:**
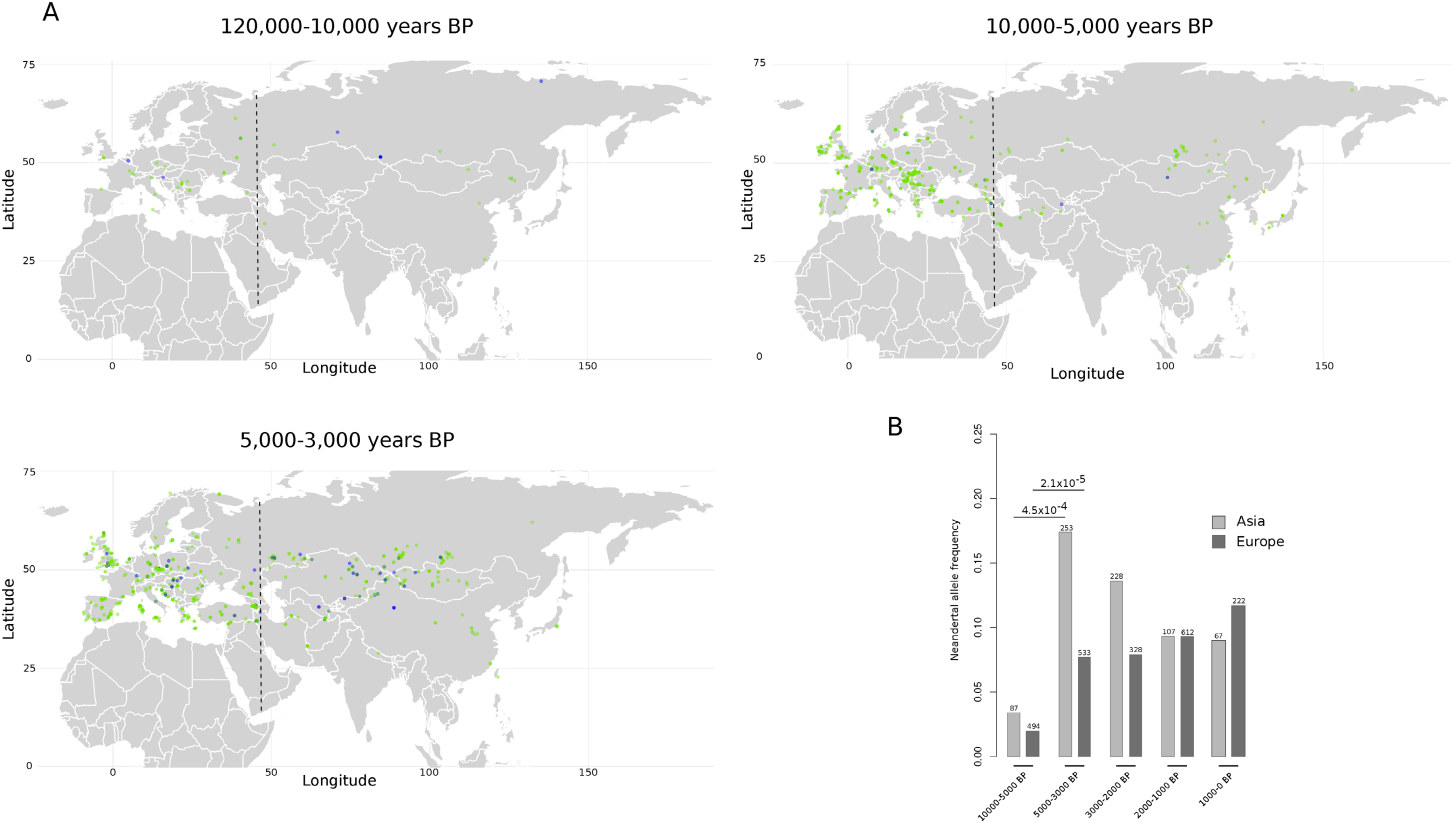
Frequency of the rs10488631 variant across time and space. (A) Geographic distribution of the Neanderthal (blue) and ancestral (green) alleles in representative time periods. The hatched line represents the boundary between Europe and Asia (45° longitude). (B) Frequency of the Neanderthal allele in Europe and Asia in 5 time periods. Significant differences between consecutive time periods are indicated (*p*‐values from proportion tests). The number of analysed individuals in each time period and continent is indicated above each bar.

In European and Asian populations, the rs12531711 SNP is in full LD with another NIM (rs10488631, *D*′ = 1, *R* ^2^ = 1), also associated with PBC and other autoimmune diseases [35] (Figure 2A). To explore the dynamics of the Neanderthal haplotype through time, we took advantage of the fact that rs10488631 is covered in genotype data from a large collection of ancient DNA samples. Most of such samples derive from individuals that lived in Europe and Asia. For this reason and given that interbreeding between anatomically modern humans and archaic hominins occurred in Eurasia [3, 32], we limited analyses to populations in these two continents. In the time frame ranging from 120 000 to 10 000 years BP, data are sparse, with only 51 individuals having information for the rs10488631 variant. Among these, 11 carry the Neanderthal‐derived allele (rs10488631‐C): eight individuals are Neanderthals or Denisovans (another group of archaic hominins related to Neanderthals), one is the Ust’‐Ishim sample (from Siberia), which was shown to carry longer segments of Neanderthal ancestry than those observed in present‐day individuals and the remaining two are individuals sampled in Russia [36]. Starting from 10 000 years BP, data are more abundant and we thus divided the genomes based on their estimated dating to analyse the frequency of rs10488631 alleles over time (Figure 3A and Figure 3B). Results indicated that, in both Europe and Asia, the frequency of the Neanderthal allele remained low until 5000 years BP (Figure 3A and Figure 3B). In the time frame between 5000 and 3000 years BP (which roughly corresponds to the Bronze Age); however, a significant increase in frequency is observed in both continents (from 2.0% to 7.7% in Europe and from 3.4% to 17.4% in Asia) (proportion test, *p*‐value< 5 × 10^−4^), followed by fluctuations of allele frequencies with no significant differences in the subsequent time frames (Figure 3B).

### 3.4 Neanderthal‐Derived Markers Do Not Contribute Overall to PBC Heritability

Studying heritability in PBC helps identify genetic factors influencing disease risk, including potential contributions from NIMs. We extracted 235 592 SNPs previously identified in the 1000 Genomes project that are likely to be Neanderthal‐derived (Neanderthal informative markers, NIMs) and examined the association of NIMs with PBC in a case–control discovery cohort containing 4615 cases and 9233 controls (CANUK). Intersecting our set of NIMs with those that were genotyped in the CANUK cohort resulted in a final list of 217 323 NIMs. We found that the heritability explained by NIMs is not significantly different (heritability enrichment of NIMs estimated at 0.11, with standard deviation of 0.42) from that of non‐NIM SNPs. There is no evidence of NIM heritability (*h* ^2^ = 0.0019, SE = 0.0075) and no evidence for either enrichment or depletion in NIM heritability.

## 4 Discussion

In this study, we confirm and extend previous findings identifying the Neanderthal‐derived variants rs12531711 on chromosome 7 as associated with increased risk of PBC. This variant, originally reported by Cordell et al. [19], resides on an introgressed haplotype that also includes rs10488631 and exhibits notable population‐level variation in frequency. Our analysis reveals that the frequency of this Neanderthal‐derived haplotype remained low in both Europe and Asia until approximately 5000 years PB, followed by a marked increase during the Bronze Age and subsequent stabilization. Functionally, rs12531711 appears to influence the expression of *TNPO3* and *IRF5*, two genes involved in key immune regulatory pathways. *TNPO3* and *IRF5* genes play distinct roles in immune responses, with effects on both viral infections and autoimmunity. *TNPO3* encodes the Transportin‐3 protein, involved in the nucleocytoplasmic transport of splicing factors [37]. Several studies showed that Transportin‐3 is also involved in the uncoating and nucleus entry of several viruses, including HIV, Rous sarcoma virus and influenza A virus [38, 39, 40, 41, 42]. Indeed, lymphocytes from patients with *TNPO3* mutations are significantly less susceptible to HIV‐1 infection than lymphocytes from non‐mutated controls [43]. Interestingly, the Neanderthal‐derived allele associated with PBC is associated with reduced *TNPO3* expression, suggesting that it may confer some advantage against virus infections, as is the case of several archaic alleles [9]. The Neanderthal‐derived allele is also associated with increased expression of *IRF5*, a key player in innate immune responses, involved in interferon signalling and the production of pro‐inflammatory cytokines. The altered expression of *IRF5* has been linked to several autoimmune diseases, including LES and multiple sclerosis (MS). Notably, a common LES risk haplotype (not deriving from introgression), which increases *IRF5* expression [44], contributes to the natural control of HIV infection and promotes bacterial clearance [45, 46]. It is thus possible that the Neanderthal haplotype contributes to both the risk of autoimmune disease and to increased resistance to infection, again in line with previous findings on the legacy of archaic alleles [2].

Because admixture between anatomically modern humans and Neandertals is estimated to have occurred between 40 000 and 54 000 years BP [3, 4, 5, 6], we leveraged the availability of thousands of ancient human genomes to follow the fate of the Neanderthal haplotype through time. Our data indicate that, both in Europe and in Asia, the frequency of the introgressed haplotype remained low until 5000 years BP, strongly increased during the Bronze Age and then stabilized. It is generally considered that the transition from a hunter‐gatherer lifestyle to agriculture and pastoralism during the Neolithic (starting around 10 000 years ago) was accompanied by changes in mobility, by the close contact with domesticated animals and by increased population density, which probably facilitated exposure to pathogens and the emergence of zoonoses [47, 48]. The Bronze Age witnessed the emergence of the first large cities [49], which may have provided the critical community size for the spread of several epidemic pathogens. Indeed, this is the time frame in which *Yersinia pestis* diverged from *Yersinia pseudotuberculosis* and evolved into the deadly pathogen causing bubonic plague [50]. Human‐adapted *Salmonella enterica* also emerged during the Bronze Age [51], as well as another epidemic pathogen, variola virus, the causative agent of smallpox [52]. It is thus possible that this changed epidemiological context favoured the spread of the Neanderthal haplotype, which may confer some protection against infections. However, the Bronze Age was also characterized by massive migrations and demographic changes in Eurasia, mainly contributed by the spread of Steppe herders from the Pontic‐Caspian area (Yamnaya expansion) [53]. The Yamnaya ancestry had a considerable impact on the genetic makeup of the Bronze Age European populations. Thus, a non‐necessarily alternative possibility is that the frequency increase of the Neanderthal haplotype resulted from demographic events.

Although the identified Neanderthal haplotype shows a significant genome‐wide association with PBC, we found that NIMs do not markedly contribute to the heritability of the disease. This observation might reflect the multifactorial nature of heritability estimates, which are influenced by both genetic and environmental factors. The complexity of genetic architecture, involving numerous small effect size variants, may dilute the detectable contribution of specific alleles, such as those of Neanderthal origin, to overall disease heritability.

## 5 Conclusions and Future Directions

Given the significant association of rs12531711 with PBC and other autoimmune diseases, future studies should clarify its functional role and potential impact on‐host pathogen interactions, as infection and autoimmunity are increasingly linked. Analyses in diverse populations, combined with ancient genomes, may also illuminate the evolutionary dynamics of the Neanderthal haplotype. While the overall contribution of Neanderthal variants to PBC heritability is likely small, their persistence and disease associations underscore the complex legacy of human evolution in shaping modern health.

## Author Contributions

A.G., P.I., R.A., S.R. initiated and coordinated the study. A.G., H.W., M.S. designed and analysed data. A.G., H.W., M.S. performed computational analyses. H.W., M.S., C.C. designed the figures. C.C., A.G. wrote the manuscript with edits from all the authors.

## Funding

This study was partially supported by NIH R01 DK126691 (KNL). A.G. and P.I. declare that this study was supported by the Italian MUR PNRR PE06 ‘HEAL ITALIA – Health Extended Alliance for Innovative Therapies, Advanced Lab‐research and Integrated Approaches of Precision Medicine’—Spoke 4—Precision Diagnostics, Italian MUR PRIN 2022 PNRR P2022H7JYZ ‘Non‐invasive biological and molecular characterization of autoimmune liver diseases and variant syndromes’ and Bando Giovani ‘Early Career Award’ FRRB—CUP H53C24000630002 ‘AIVAR—Artificial Intelligence‐Enhanced Diagnosis of PBC‐AIH Variant Syndrome’.

## Ethics Statement

The authors have nothing to report.

## Conflicts of Interest

The authors declare no conflicts of interest.

## Supporting information


**Figure S1:** Q‐Q plots for GWAS results with and without nimpc adjustment. Genome‐wide quantile‐quantile (Q‐Q) plots comparing observed versus expected ${-\log}_{10}$(*p*‐values) for association statistics. The left panel shows the Q‐Q plot for the GWAS analysis with nimpc correction, while the right panel presents the results without the adjustment. In both plots, the majority of *p*‐values align closely with the null expectation (red line), indicating appropriate control of type I error. However, the nimpc‐adjusted analysis (left) shows a slightly tighter adherence to the null in the lower tail, suggesting improved calibration. Both plots show a strong deviation in the upper tail, indicating the presence of true genetic associations. The nimpc adjustment may reduce inflation and increase confidence in the top signals by accounting for potential confounders or improving model fit.
**Figure S2:** Functional annotation of rs12531711 using RegulomeDB. The table reports the searched genomic coordinates, the number of overlapping regulatory peaks, the assigned RegulomeDB rank, the corresponding score and the direct link to the RegulomeDB query.

## Data Availability

The data that support the findings of this study are available from the corresponding author upon reasonable request.
