# Supplementary material for: A Genetic Risk Variant Associated With the Risk of Primary Biliary Cholangitis Is Inherited From Neanderthals
Source: Liver Int. 2026 Sep 22;46(10):e70868. doi: 10.1111/liv.70868 (PMC13598353; doi:10.1111/liv.70868)
Supplement: Supplementary file 1 [file LIV-46-0-s001.docx]

# **SUPPLEMENTARY**

Collaborators:

**Italian PBC Genetics Study Group**

Andrea Affronti^21^, Maurizia Brunetto^22^, Barbara Coco^22^, Giancarlo Spinzi^23^, Gianfranco Elia^24^, Carlo Ferrari^24^, Ana Lleo^17^, Luigi Muratori^25^, Paolo Muratori^25^, Piero Portincasa^26^, Agostino Colli^27^, Savino Bruno^28^, Guido Colloredo^29^, Francesco Azzaroli^30^, Pietro Andreone^30^, Maria Consiglia Bragazzi^31^, Domenico Alvaro^31^, Vincenzo Cardinale^31^, Nora Cazzagon^32^, Annarosa Floreani^32^, Cristina Rigamonti^33^, Floriano Rosina^34^, Marco Carbone^1,2^, Laura Cristoferi^1,2^, Marco Festa^1,2^, Eugenia Nofit^1,2^, Miki Scaravaglio^1,2^, Daphne D’Amato^1,2^, Federica Malinverno^1,2^, Pietro Lampertico^35^, Francesca Donato^35^, Stefano Fagiuoli^36^, Piero L. Almasio^37^, Edoardo Giannini^38^, Carmela Cursaro^39^, Massimo Colombo^40^, Luca Valenti^41^, Luca Miele^56^, Angelo Andriulli^57^, Grazia A. Niro^57^, Ignazio Grattagliano^42^, Lorenzo Morini^43^, Giovanni Casella^44^, Maria Vinci^45^, Pier Maria Battezzati^46^, Andrea Crosignani^46^, Massimo Zuin^46^, Alberto Mattalia^47^, Vincenza Calvaruso^37^, Silvia Colombo^48^, Antonio Benedetti^49^, Marco Marzioni^49^, Andrea Galli^50^, Fabio Marra^50^, Mirko Tarocchi^50^, Antonio Picciotto^51^, Filomena Morisco^52^, Luca Fabris^53^, Lory Saveria Crocè^54^, Claudio Tiribelli^54^, Pierluigi Toniutto^55^, Mario Strazzabosco^58^

**UK-PBC Consortium**

James J Fryett^4^, Rebecca Darlay^4^, Steven Flack^59^, Ann Spicer^59^, Victoria L Mulcahy^59^, Richard Sturgess^60^, Christopher Healey^61^, Andrew Yeoman^62^, Anton Vj Gunasekera^63^, Paul Kooner^64^, Kapil Kapur^65^, V Sathyanarayana^66^, Yiannis Kallis^66^, Javaid Subhani^67^, Rory Harvey^68^, Roger McCorry^69^, Paul Rooney^69^, David Ramanaden^70^, Richard Evans^70^, Thiriloganathan Mathialahan^70^, Jaber Gasem^70^, Christopher Shorrock^71^, Mahesh Bhalme^72^, Paul Southern^73^, Jeremy A Tibble^74^, David A Gorard^75^, Susan Jones^76^, Brijesh Srivastava^77^, Matthew R Foxton^78^, Carole E Collins^78^, David Elphick^79^, Mazn Karmo^80^, Francisco Porras-Perez^81^, Michael Mendall^82^, Tom Yapp^83^, Minesh Patel^83^, Roland Ede^84^, Joanne Sayer^85^, James Jupp^86^, Neil Fisher^87^, Martyn J Carter^88^, Konrad Koss^89^, Jayshri Shah^90^, Andrzej Piotrowicz^90^, Glyn Scott^90^, Charles Grimley^91^, Ian R Gooding^92^, Simon Williams^92^, Judith Tidbury^93^, Guan Lim^94^, Kuldeep Cheent^95^, Sass Levi^95^, Dina Mansour^96^, Matilda Beckley^97^, Coral Hollywood^98^, Terry Wong^99^, Richard Marley^99^, John Ramage^100^, Harriet M Gordon^100^, Jo Ridpath^101^, Theodore Ngatchu^102^, Vijay Paul Bob Grover^103^, Ray G Shidrawi^104^, George Abouda^105^, L Corless^105^, Mark Narain^106^, Ian Rees^106^, Ashley Brown^107^, Simon Taylor-Robinson^107^, Joy Wilkins^108^, Leonie Grellier^108^, Paul Banim^109^, Debasish Das^110^, Michael A Heneghan^111^, Howard Curtis^111^, Helen C Matthews^112^, Faiyaz Mohammed^113^, Mark Aldersley^114^, Raj Srirajaskanthan^115^, Giles Walker^115^, Alistair McNair^115^, Amar Sharif^116^, Sambit Sen^117^, George Bird^118^, Martin I Prince^119^, Geeta Prasad^119^, Paul Kitchen^120^, Adrian Barnardo^120^, Chirag Oza^121^, Nurani N Sivaramakrishnan^122^, Prakash Gupta^123^, Amir Shah^124^, Chris Dj Evans^125^, Subrata Saha^126^, Katharine Pollock^127^, Peter Bramley^128^, Ashis Mukhopadhya^129^, Stephen T Barclay^130^, Natasha McDonald^131^, Andrew J Bathgate^132^, Kelvin Palmer^132^, John F Dillon^133^, Simon M Rushbrook^134^, Robert Przemioslo^135^, Chris McDonald^136^, Andrew Millar^137^, Cheh Tai^137^, Stephen Mitchell^138^, Jane Metcalf^138^, Syed Shaukat^139^, Mary Ninkovic^139^, Udi Shmueli^140^, Andrew Davis^141^, Asifabbas Naqvi^142^, Tom Jw Lee^143^, Stephen Ryder^144^, Jane Collier^145^, Howard Klass^146^, Matthew E Cramp^147^, Nichols Sharer^148^, Richard Aspinall^149^, Deb Ghosh^150^, Andrew C Douds^151^, Jonathan Booth^152^, Earl Williams^153^, Hyder Hussaini^154^, John Christie^155^, Steven Mann^156^, Douglas Thorburn^156^, Aileen Marshall^156^, Imran Patanwala^157^, Aftab Ala^158^, Julia Maltby^159^, Ray Matthew^160^, Chris Corbett^160^, Sam Vyas^161^, Saket Singhal^162^, Dermot Gleeson^163^, Sharat Misra^164^, Jeff Butterworth^165^, Keith George^166^, Tim Harding^167^, Andrew Douglass^168^, Harriet Mitchison^169^, Simon Panter^169^, Jeremy Shearman^170^, Gary Bray^171^, Michael Roberts^172^, Graham Butcher^172^, Daniel Forton^173^, Zahid Mahmood^174^, Matthew Cowan^175^, Debashis Das^175^, Chin Lye Ch'ng^176^, Mesbah Rahman^176^, Gregory C A Whatley^177^, Emma Wesley^178^, Aditya Mandal^179^, Sanjiv Jain^179^, Stephen P Pereira^180^, Mark Wright^181^, Palak Trivedi^182^, Fiona H Gordon^183^, Esther Unitt^184^, Altaf Palejwala^185^, Andrew Austin^185^, Vishwaraj Vemala^186^, Allister Grant^186^, Andrew D Higham^187^, Alison Brind^188^, Ray Mathew^188^, Mark Cox^189^, Subramaniam Ramakrishnan^190^, Alistair King^191^, Simon Whalley^192^, Jocelyn Fraser^193^, S J Thomson^193^, Andrew Bell^194^, Voi Shim Wong^195^, Richard Kia^196^, Ian Gee^197^, Richard Keld^198^, Rupert Ransford^199^, James Gotto^200^, Charles Millson^201^

**Canadian-US PBC Consortium**

Andrew Mason^202^, Catherine Vincent^203^, Gang Xie^204^, Jinyi Zhang^204^

**Additional affiliations**

21.	Azienda Ospedaliera Ospedali Riuniti Villa Sofia-Cervello, Palermo, Italy

22.	Azienda Ospedaliera Universitaria Pisana, Pisa, Italy

23.	Azienda Ospedaliera Valduce, Como, Italy

24.	Azienda Ospedaliero-Universitaria di Parma, Parma, Italy

25.	Department of Clinical Medicine, University of Bologna, Bologna, Italy

26.	Department of Interdisciplinary Medicine, University Medical School, Bari, Italy

27.	Department of Internal Medicine, AO Provincia di Lecco, Lecco, Italy

28.	Department of Internal Medicine, Ospedale Fatebenefratelli e Oftalmico, Milan, Italy

29.	Department of Internal Medicine, San Pietro Hospital, Ponte San Pietro, Bergamo, Italy

30.	Department of Medical and Surgical Sciences (DIMEC), University of Bologna, Bologna, Italy

31.	Department of Medico-Surgical Sciences and Biotechnologies, Sapienza University of Rome, Rome, Italy

32.	Department of Surgery, Oncology and Gastroenterology, University of Padua, Padua, Italy

33.	Department of Translational Medicine, Università del Piemonte Orientale (UPO), Novara, Italy

34.	Division of Gastroenterology & Hepatology, Center for Predictive Medicine, Gradenigo Hospital, Turin, Italy

35.	Division of Gastroenterology and Hepatology, Fondazione IRCCS Ca' Granda Ospedale Maggiore Policlinico, Milan, Italy

36.	Gastroenterologia Epatologia e Trapiantologia, Papa Giovanni XXIII Hospital, Bergamo, Italy

37.	Gastroenterology & Hepatology Unit, Biomedical Department of Internal and Specialistic Medicine (Di.Bi.M.I.S.), University of Palermo, Palermo, Italy

38.	Gastroenterology Unit, Department of Internal Medicine, Policlinico San Martino, University of Genoa, Genoa, Italy

39.	Hepatology Unit, Department of Medical and Surgical Sciences, University Hospital of Bologna, Bologna, Italy

40.	Liver Center, IRCCS San Raffaele Hospital, Milan, Italy

41.	Department of Pathophysiology and Transplantation, Università degli Studi di Milano, Milan, Italy

42.	Italian College of General Practitioners, ASL Bari, Bari, Italy

43.	Magenta Hospital, Magenta, Italy

44.	Medical Department, Desio Hospital, Desio, Italy

45.	Ospedale Niguarda, Milan, Italy

46.	San Paolo Hospital Medical School, Università di Milano, Milan, Italy

47.	Santa Croce e Carle Hospital, Cuneo, Italy

48.	Treviglio Hospital, Treviglio, Italy

49.	Università Politecnica delle Marche, Ancona, Italy

50.	University of Florence, Florence, Italy

51.	University of Genoa, Genoa, Italy

52.	University of Naples Federico II, Naples, Italy

53.	University of Padua, Padua, Italy

54.	University of Trieste and Fondazione Italiana Fegato (FIF), Trieste, Italy

55.	University of Udine, Udine, Italy

56.	Fondazione Policlinico Universitario A. Gemelli IRCCS, Università Cattolica del Sacro Cuore, Rome, Italy

57.	Fondazione IRCCS Casa Sollievo della Sofferenza, San Giovanni Rotondo, Italy

58.	Yale University, New Haven, Connecticut, USA

59.	Academic Department of Medical Genetics, University of Cambridge, Cambridge, United Kingdom

60.	Aintree University Hospital, Aintree University Hospitals NHS Foundation Trust, United Kingdom

61.	Airedale General Hospital, Airedale NHS Foundation Trust, United Kingdom

62.	Nevill Hall Hospital, Royal Gwent Hospital, Ysbyty Ystrad Fawr, Aneurin Bevan University Health Board, United Kingdom

63.	Ashford Hospital, St Peter's Hospital, Ashford and St Peter's NHS Foundation Trust, United Kingdom

64.	Queen's Hospital, King George Hospital, Barking, Havering and Redbridge University Hospitals NHS Trust, United Kingdom

65.	Barnsley Hospital, Barnsley Hospital NHS Foundation Trust, United Kingdom

66.	Newham University Hospital, St Bartholomew's Hospital, The Royal London Hospital, Whipps Cross University Hospital, Barts Health NHS Trust, United Kingdom

67.	Basildon University Hospital, Basildon and Thurrock University Hospitals NHS Foundation Trust, United Kingdom

68.	Bedford Hospital, Bedford Hospitals NHS Trust, United Kingdom

69.	Royal Victoria Hospital, Belfast Health and Social Care Trust, United Kingdom

70.	Glan Clwyd Hospital, Llandudno General Hospital, Wrexham Maelor Hospital, Ysbyty Gwynedd, Betsi Cadwaladr University Health Board, United Kingdom

71.	Blackpool Victoria Hospital, Blackpool Teaching Hospitals NHS Foundation Trust, United Kingdom

72.	Royal Bolton Hospital, Bolton NHS Foundation Trust, United Kingdom

73.	Bradford Royal Infirmary, Bradford Teaching Hospitals NHS Foundation Trust, United Kingdom

74.	Royal Sussex County Hospital, Brighton and Sussex University Hospitals NHS Trust, United Kingdom

75.	Amersham Hospital, Stoke Mandeville Hospital, Wycombe Hospital, Buckinghamshire Healthcare NHS Trust, United Kingdom

76.	Calderdale Royal Hospital, Huddersfield Royal Infirmary, Calderdale and Huddersfield NHS Trust, United Kingdom

77.	University Hospital Llandough, University Hospital of Wales, Cardiff and Vale University Health Board, United Kingdom

78.	Chelsea and Westminster Hospital, West Middlesex University Hospital, Chelsea & Westminster NHS Foundation Trust, United Kingdom

79.	Chesterfield Royal Hospital, Chesterfield Royal Hospital NHS Foundation Trust, United Kingdom

80.	Countess of Chester Hospital, Countess of Chester Hospital NHS Foundation Trust, United Kingdom

81.	Darlington Memorial Hospital, University Hospital of North Durham, County Durham and Darlington NHS Foundation Trust, United Kingdom

82.	Croydon University Hospital, Purley War Memorial Hospital, Croydon Health Services NHS Trust, United Kingdom

83.	Princess of Wales Hospital, Prince Charles Hospital, Royal Glamorgan Hospital, Ysbyty Cwm Cynon, Cwm Taf Morgannwg University Health Board, United Kingdom

84.	Darent Valley Hospital, Queen Mary's Hospital Sidcup, Dartford and Gravesham NHS Trust, United Kingdom

85.	Bassetlaw Hospital, Doncaster Royal Infirmary, Doncaster and Bassetlaw Hospitals NHS Foundation Trust, United Kingdom

86.	Dorset County Hospital, Dorset County Hospital NHS Foundation Trust, United Kingdom

87.	Russells Hall Hospital, Dudley Group of Hospitals NHS Trust, United Kingdom

88.	Lister Hospital, Queen Elizabeth II Hospital, East and North Hertfordshire NHS Trust, United Kingdom

89.	Macclesfield District General Hospital, East Cheshire NHS Trust, United Kingdom

90.	Buckland Hospital, Kent and Canterbury Hospital, Queen Elizabeth The Queen Mother Hospital, William Harvey Hospital Ashford, East Kent Hospitals University NHS Foundation Trust, United Kingdom

91.	Burnley General Hospital, Royal Blackburn Hospital, East Lancashire Hospitals NHS Trust, United Kingdom

92.	Colchester General Hospital, Ipswich Hospital, East Suffolk and North Essex NHS Foundation Trust, United Kingdom

93.	Conquest Hospital, Eastbourne District General Hospital, East Sussex Healthcare NHS Trust, United Kingdom

94.	Epsom General Hospital, Epsom and St Helier University Hospitals NHS Trust, United Kingdom

95.	Frimley Park Hospital, Heatherwood Hospital, Wexham Park Hospital, Frimley Health NHS Foundation Trust, United Kingdom

96.	Queen Elizabeth Hospital, Gateshead Health NHS Foundation Trust, United Kingdom

97.	George Eliot Hospital, George Eliot Hospital NHS Trust, United Kingdom

98.	Cheltenham General Hospital, Gloucestershire Royal Hospital, Gloucestershire Hospitals NHS Foundation Trust, United Kingdom

99.	Guy's Hospital, St Thomas' Hospital, Guy's and St Thomas' NHS Foundation Trust, United Kingdom

100.	Basingstoke and North Hampshire Hospital, Royal Hampshire County Hospital, Hampshire Hospitals NHS Foundation Trust, United Kingdom

101.	Harrogate District Hospital, Harrogate and District Foundation Trust, United Kingdom

102.	Good Hope Hospital, Heartlands Hospital, Solihull Hospital, Heart of England NHS Foundation Trust, United Kingdom

103.	Hillingdon Hospital, Hillingdon Hospitals NHS Foundation Trust, United Kingdom

104.	Homerton University Hospital, Homerton University Hospital NHS Foundation Trust, United Kingdom

105.	Castle Hill Hospital, Hull Royal Infirmary, Hull University Teaching Hospitals NHS Trust, United Kingdom

106.	Bronglais Hospital, Prince Philip Hospital, Withybush General Hospital, Glangwili General Hospital, Hywel Dda University Health Board, United Kingdom

107.	Charing Cross Hospital, Hammersmith Hospital, St Mary's Hospital, Imperial College, Imperial College Healthcare NHS Trust, United Kingdom

108.	St Mary's Hospital, Isle of Wight, Isle of Wight NHS Trust, United Kingdom

109.	James Paget University Hospital, James Paget University Hospitals NHS Foundation Trust, United Kingdom

110.	Kettering General Hospital, Kettering General Hospital NHS Foundation Trust, United Kingdom

111.	King's College Hospital, Beckenham Beacon, Orpington Hospital, Princess Royal University Hospital, King's College Hospital NHS Foundation Trust, United Kingdom

112.	Kingston Hospital, Kingston Hospital NHS Foundation Trust, United Kingdom

113.	Chorley and South Ribble Hospital, Royal Preston Hospital, Lancashire Teaching Hospitals NHS Foundation Trust, United Kingdom

114.	St James's University Hospital, Leeds Teaching Hospitals NHS Trust, United Kingdom

115.	University Hospital Lewisham, Queen Elizabeth Hospital, Lewisham and Greenwich NHS Trust, United Kingdom

116.	Central Middlesex Hospital, Northwick Park and St Mark's Hospitals, London North West Healthcare NHS Trust, United Kingdom

117.	Luton and Dunstable University Hospital, Luton and Dunstable University Hospital NHS Foundation Trust, United Kingdom

118.	Maidstone Hospital, Tunbridge Wells Hospital, Maidstone and Tunbridge Wells NHS Trust, United Kingdom

119.	Manchester Royal Infirmary, Trafford General Hospital, Wythenshawe Hospital, Manchester University NHS Foundation Trust, United Kingdom

120.	Medway Maritime Hospital, Medway NHS Foundation Trust, United Kingdom

121.	Broomfield Hospital, St Peter's Hospital, Mid-Essex Hospitals NHS Trust, United Kingdom

122.	Dewsbury and District Hospital, Mid Yorkshire Hospitals NHS Trust, United Kingdom

123.	Milton Keynes Hospital, Milton Keynes Hospital NHS Foundation Trust, United Kingdom

124.	University Hospital Crosshouse, NHS Ayrshire & Arran, United Kingdom

125.	Borders General Hospital, NHS Borders, United Kingdom

126.	Dumfries and Galloway Royal Infirmary, NHS Dumfries & Galloway, United Kingdom

127.	Queen Margaret Hospital, Victoria Hospital, NHS Fife, United Kingdom

128.	Falkirk Community Hospital, Forth Valley Royal Hospital, Stirling Community Hospital, NHS Forth Valley, United Kingdom

129.	Aberdeen Royal Infirmary, Dr Gray's Hospital, NHS Grampian, United Kingdom

130.	Gartnavel General Hospital, Glasgow Royal Infirmary, Inverclyde Royal Hospital, Royal Alexandra Hospital, Southern General Hospital, Victoria Infirmary, NHS Greater Glasgow and Clyde, United Kingdom

131.	Hairmyres Hospital, Monklands Hospital, Wishaw General Hospital, NHS Lanarkshire, United Kingdom

132.	Royal Infirmary of Edinburgh, St John's Hospital, Western General Hospital, NHS Lothian, United Kingdom

133.	Ninewells Hospital, NHS Tayside, United Kingdom

134.	Norfolk and Norwich University Hospital, Norfolk and Norwich University Hospitals NHS Foundation Trust, United Kingdom

135.	Southmead Hospital, North Bristol NHS Trust, United Kingdom

136.	Cumberland Infirmary, West Cumberland Hospital, North Cumbria University Hospitals NHS Foundation Trust, United Kingdom

137.	North Middlesex Hospital, North Middlesex University Hospital NHS Trust, United Kingdom

138.	University Hospital of Hartlepool, University Hospital of North Tees, North Tees and Hartlepool NHS Foundation Trust, United Kingdom

139.	Hinchingbrooke Hospital, Peterborough City Hospital, Stamford & Rutland Hospital, North West Anglia NHS Foundation Trust, United Kingdom

140.	Northampton General Hospital, Northampton General Hospital NHS Trust, United Kingdom

141.	North Devon District Hospital, Northern Devon Healthcare NHS Trust, United Kingdom

142.	Diana Princess of Wales Hospital, Scunthorpe General Hospital, Northern Lincolnshire and Goole NHS Foundation Trust, United Kingdom

143.	North Tyneside General Hospital, Northumbria Healthcare NHS Foundation Trust, United Kingdom

144.	Nottingham City Hospital, Queen's Medical Centre, NIHR Nottingham Biomedical Research Centre at Nottingham University Hospitals NHS Trust, United Kingdom

145.	John Radcliffe Hospital, Oxford University Hospitals NHS Trust, United Kingdom

146.	Fairfield General Hospital, North Manchester General Hospital, Rochdale Infirmary, The Royal Oldham Hospital, Pennine Acute Hospitals NHS Trust, United Kingdom

147.	Derriford Hospital, University Hospitals Plymouth NHS Trust, United Kingdom

148.	Poole Hospital, Poole Hospital NHS Foundation Trust, United Kingdom

149.	Queen Alexandra Hospital, Portsmouth Hospitals NHS Trust, United Kingdom

150.	St Margaret's Hospital, The Princess Alexandra Hospital, Princess Alexandra Hospital NHS Trust, United Kingdom

151.	The Queen Elizabeth Hospital King's Lynn, King's Lynn NHS Foundation Trust, United Kingdom

152.	Royal Berkshire Hospital, Royal Berkshire NHS Foundation Trust, United Kingdom

153.	Royal Bournemouth Hospital, Royal Bournemouth and Christchurch Hospitals NHS Foundation Trust, United Kingdom

154.	Royal Cornwall Hospital, Royal Cornwall Hospitals NHS Trust, United Kingdom

155.	Royal Devon and Exeter Hospital, Royal Devon and Exeter NHS Foundation Trust, United Kingdom

156.	Barnet and Chase Farm Hospitals, The Royal Free Hospital, Royal Free London NHS Foundation Trust, United Kingdom

157.	Royal Liverpool University Hospital, Royal Liverpool and Broadgreen University Hospitals NHS Trust, United Kingdom

158.	Royal Surrey County Hospital, Royal Surrey County Hospital NHS Foundation Trust, United Kingdom

159.	Royal United Bath Hospital, Royal United Hospitals Bath NHS Foundation Trust, United Kingdom

160.	Cannock Chase Hospital, New Cross Hospital, Royal Wolverhampton NHS Trust, United Kingdom

161.	Salisbury District Hospital, Salisbury NHS Foundation Trust, United Kingdom

162.	Sandwell General Hospital, Sandwell & West Birmingham Hospitals NHS Trust, United Kingdom

163.	Northern General Hospital, Royal Hallamshire Hospital, Sheffield Teaching Hospitals NHS Foundation Trust, United Kingdom

164.	King's Mill Hospital, Newark Hospital, Sherwood Forest Hospitals NHS Foundation Trust, United Kingdom

165.	Princess Royal Hospital, Royal Shrewsbury Hospital, Shrewsbury and Telford Hospital NHS Trust, United Kingdom

166.	Torbay Hospital, Torbay and South Devon NHS Foundation Trust, United Kingdom

167.	Lagan Valley Hospital, Ulster Hospital, South Eastern Health and Social Care Trust, United Kingdom

168.	Friarage Hospital, The James Cook University Hospital, South Tees Hospitals NHS Foundation Trust, United Kingdom

169.	Sunderland Royal Hospital, South Tyneside District Hospital, South Tyneside and Sunderland NHS Foundation Trust, United Kingdom

170.	Warwick Hospital, South Warwickshire NHS Foundation Trust, United Kingdom

171.	Southend University Hospital, Southend University Hospital NHS Foundation Trust, United Kingdom

172.	Ormskirk District General Hospital, Southport and Formby District General Hospital, Southport & Ormskirk Hospital NHS Trust, United Kingdom

173.	St George's Hospital, St George's University Hospitals NHS Foundation Trust, United Kingdom

174.	Stepping Hill Hospital, Stockport NHS Foundation Trust, United Kingdom

175.	East Surrey Hospital, Surrey and Sussex Healthcare NHS Trust, United Kingdom

176.	Morriston Hospital, Singleton Hospital, Neath Port Talbot Hospital, Swansea Bay University Health Board, United Kingdom

177.	Tameside General Hospital, Tameside and Glossop Integrated Care NHS Trust, United Kingdom

178.	Musgrove Park Hospital, Taunton and Somerset NHS Foundation Trust, United Kingdom

179.	Lincoln County Hospital, Pilgrim Hospital Boston, United Lincolnshire Hospitals NHS Trust, United Kingdom

180.	University College Hospital, University College London Hospitals NHS Foundation Trust, United Kingdom

181.	Southampton General Hospital, University Hospital Southampton NHS Foundation Trust, United Kingdom

182.	The Queen Elizabeth Hospital, University Hospitals Birmingham NHS Foundation Trust, United Kingdom

183.	Bristol Royal Infirmary, University Hospitals Bristol NHS Foundation Trust, United Kingdom

184.	University Hospital, Coventry, University Hospital Coventry and Warwickshire NHS Trust, United Kingdom

185.	Queen's Hospital, Royal Derby Hospital, University Hospitals of Derby and Burton NHS Foundation Trust, United Kingdom

186.	Glenfield Hospital, Leicester General Hospital, Leicester Royal Infirmary, University Hospitals of Leicester NHS Trust, United Kingdom

187.	Royal Lancaster Infirmary, University Hospitals of Morecambe Bay NHS Foundation Trust, United Kingdom

188.	Royal Stoke University Hospital, County Hospital, University Hospitals of North Midlands NHS Trust, United Kingdom

189.	Walsall Manor Hospital, Walsall Healthcare NHS Trust, United Kingdom

190.	Warrington Hospital, Warrington and Halton Hospitals NHS Foundation Trust, United Kingdom

191.	Hemel Hempstead General Hospital, St Albans City Hospital, Watford General Hospital, West Hertfordshire Hospitals NHS Trust, United Kingdom

192.	West Suffolk Hospital, West Suffolk NHS Foundation Trust, United Kingdom

193.	St Richard's Hospital, Worthing Hospital, Western Sussex Hospitals NHS Foundation Trust, United Kingdom

194.	Weston General Hospital, Weston Area Health Trust, United Kingdom

195.	The Whittington Hospital, Whittington Hospital NHS Trust, United Kingdom

196.	Arrowe Park Hospital, Victoria Central Hospital, Wirral University Teaching Hospital NHS Foundation Trust, United Kingdom

197.	Alexandra Hospital, Kidderminster Hospital and Treatment Centre, Worcestershire Royal Hospital, Worcestershire Acute Hospitals NHS Trust, United Kingdom

198.	Royal Albert Edward Infirmary, Wrightington, Wigan and Leigh NHS Trust, United Kingdom

199.	The County Hospital, Wye Valley NHS Trust, United Kingdom

200.	Yeovil District Hospital, Yeovil District Hospital NHS Foundation Trust, United Kingdom

201.	Bridlington Hospital, Scarborough Hospital, The York Hospital, York Teaching Hospitals NHS Foundation Trust, United Kingdom

202.	Dept. of Medicine, University of Alberta, Edmonton, Alberta, Canada

203.	Universite de Montreal Hospital Centre, Saint-Luc Hospital, Montreal, Quebec, Canada

204.	Lunenfeld Tanenbaum Research Institute, Toronto, Ontario, Canada

# **SUPPLEMENTARY FIGURES**

**Supplementary Fig. 1 | Q-Q plots for GWAS results with and without nimpc adjustment.** Genome-wide quantile-quantile (Q-Q) plots comparing observed versus expected ${-log}_{10}$(p-values) for association statistics. The left panel shows the Q-Q plot for the GWAS analysis with nimpc correction, while the right panel presents the results without the adjustment. In both plots, the majority of p-values align closely with the null expectation (red line), indicating appropriate control of type I error. However, the nimpc-adjusted analysis (left) shows a slightly tighter adherence to the null in the lower tail, suggesting improved calibration. Both plots show a strong deviation in the upper tail, indicating the presence of true genetic associations. The nimpc adjustment may reduce inflation and increase confidence in the top signals by accounting for potential confounders or improving model fit.

| **Variant** | **Searched coordinates** | **Peaks** | **Rank** | **Score** | **Link** |
| --- | --- | --- | --- | --- | --- |
| rs12531711 | chr7:128977411-128977412 | 157 | 1f | 0.55324 | https://regulomedb.org/regulome-search?regions=chr7%3A128977411-128977412&genome=GRCh38 |

**Supplementary Fig. 2 | Functional annotation of rs12531711 using RegulomeDB.** The table reports the searched genomic coordinates, the number of overlapping regulatory peaks, the assigned RegulomeDB rank, the corresponding score and the direct link to the RegulomeDB query.
